# Effect of Antenatal Exercises, Including Yoga, on the Course of Labor, Delivery and Pregnancy: A Retrospective Study

**DOI:** 10.3390/ijerph17155274

**Published:** 2020-07-22

**Authors:** Yogyata Wadhwa, Ahmad H. Alghadir, Zaheen A. Iqbal

**Affiliations:** 1 Health on My Mind, Gurugram, Haryana 122002, India; physio_yogyata@yahoo.co.in; 2 Rehabilitation Research Chair, College of Applied Medical Sciences, King Saud University, Riyadh 11433, Saudi Arabia; aha@ksu.edu.sa

**Keywords:** pregnancy, antenatal exercises, yoga, labor, pain, delivery, women’s health

## Abstract

*Background*: Delivering a child is a very stressful experience for women. Pregnancy and labor entail complex events that are unique to each individual female. The management of labor pain is often done using analgesics and anesthesia, which have been shown to have some side effects. More comprehensive data are needed to provide clinically significant evidence for clinicians to confidently prescribe exercises to patients. This study was done to evaluate the effect of antenatal exercises, including yoga, on the course of labor, delivery, and pregnancy outcomes. *Methods*: A retrospective study was conducted among 200 primiparous subjects (aged 20–40). A questionnaire was provided to the subjects to obtain their demographic and obstetrical information 6 weeks after delivery, and their hospital records were also assessed for further details. Based on the nature and details obtained for the antenatal exercises, subjects were divided into two groups: control and exercise. Outcome measures included the need for labor induction, self-perceived pain and perceived exertion during labor, duration and nature of the delivery, newborn infant weight, maternal weight gain, history of back pain, and post-partum recovery. The total maternal weight gain (in kilograms) was calculated from weight at 6 weeks after delivery minus the weight at 12–14 weeks of gestation. Back pain during pregnancy and self-perceived labor pain were measured using a visual analog scale (VAS). The overall perceived exertion during labor was measured using an adapted Borg scale for perceived effort. *Results*: The subjects who followed regular antenatal exercises, including yoga, had significantly lower rates of cesarean section, lower weight gain, higher newborn infant weight, lower pain and overall discomfort during labor, lower back pain throughout pregnancy, and earlier post-partum recovery compared to those who did no specific exercises or only walked during pregnancy. *Conclusions*: This retrospective study showed that regular antenatal exercises, including yoga, result in better outcomes related to the course of labor, delivery, and pregnancy. These results notably indicated that pregnant women should be active throughout pregnancy and follow a supervised exercise program that includes yoga unless contraindicated. We require further large-scale prospective studies and quasi-experimental trials to confirm the observed findings.

## 1. Introduction

Delivering a child is a very stressful experience for women, especially for the first time [1]. Pregnancy and labor entail complex events that are unique to each individual female [2]. Preparation for delivery can be effective in decreasing adverse responses during labor [1,3]. A prolonged duration of labor and other complications, such as abnormal fetal position or heart rate, can result in a cesarean section [4]. The rate of cesarean deliveries has increased in recent times, although it varies with healthcare providers and the site of delivery [5,6,7]. A cesarean section is an invasive procedure with various risks, including infection, hemorrhage, thromboembolism, and potential morbidity for the mother and the infant [8,9]. It has also become the choice for women who are afraid of pain during the course of labor and delivery [10]. However, obstetricians should restrict the option of surgical delivery to complicated cases, where the risk for the mother, her infant, or both are high [5]. In addition to reducing complications, decreasing the rate of cesarean sections would also reduce the financial burden on healthcare [11].

Management of labor pain is often done using analgesics and anesthesia, which have been shown to provide relief to mothers [12]. The usage of analgesics and anesthesia at this stage can lead to negative effects for both the mother and the infant [4,12,13]. The use of non-pharmacological methods for pain reduction can be more effective physically, psychologically, and emotionally, as well as less damaging for the mother and fetus [14,15,16].

A few studies on the effects of exercises during pregnancy have reported mixed results related to preterm labor chances, intrauterine growth retardation (IUGR), pregnancy-induced hypertension, gestational diabetes, labor pain and duration, and the risk of cesarean section [4,11,17,18,19,20,21,22]. This could be due to various limitations, including a smaller sample size, the involvement of different forms of exercises and their protocols, and failure to comply with the exercises [11,23]. More comprehensive data are needed to provide clinically significant evidence for clinicians to confidently prescribe exercises to patients.

This study was performed to observe the effects of antenatal exercises, including yoga, on the course of labor, delivery, and pregnancy outcomes. We hypothesized that females who follow antenatal exercises, including yoga, for at least three months would have better outcomes in the course of labor and delivery, maternal weight gain and newborn infant weight, post-partum recovery, back pain during pregnancy, and pain and overall discomfort during labor.

## 2. Materials and Methods

A retrospective study was conducted among 200 primiparous subjects (aged 20–40), who delivered their infant between 1 April 2018 and 30 April 2019. Subjects were excluded if they had a history of any serious illness (diabetes, hypertension, etc.) before pregnancy or any high-risk complication during pregnancy or delivery, such as fetal abnormalities and IUGR. Data for subjects who opted for a planned cesarean section were not included in the study. After considering these criteria, 158 subjects were included in this study. All subjects were informed about the aims and nature of the study and their written consent was obtained prior to data collection. This study was approved by the Ethics Research Committee in Institutional Review Board (Ref no. KSU/RRC/045/03).

A questionnaire was provided to the subjects to obtain their demographic and obstetrical information 6 weeks after delivery. This included age, education level, job details, body mass index (BMI), lifestyle before becoming pregnant, the nature and details of their antenatal exercises, need for labor induction, self-perceived labor pain and perceived exertion, duration and nature of delivery, newborn infant weight, maternal weight gain, history of back pain, and post-partum recovery (Table 1). Their hospital records were also assessed for further details.

Based on the information obtained, subjects were divided into two groups: control and exercise. Subjects who followed supervised antenatal exercises, including resistance, aerobic, yoga, pelvic floor, stretching, relaxation exercises, or a combination program with or without walking, for at least 3 months during pregnancy and for at least one (minimum half an hour) session per week were included in the exercise group. Subjects who did no specific exercises or only walked during pregnancy were included in the control group.

The total maternal weight gain (in kilograms) was calculated by weight at 6 weeks after delivery minus the weight at 12–14 weeks of gestation. Participants were requested to report their post-partum recovery based on the time after delivery that they returned to household tasks, including making the bed, sweeping/mopping/cleaning, grocery shopping, kitchen work without help, and working a job, if employed. The time to resume these activities was recorded as early (2–3 weeks) or delayed (>3 weeks).

Back pain during pregnancy and self-perceived labor pain were measured using the visual analog scale (VAS) [24]. Subjects had to rate their worst pain on a scale of 10, with 0 meaning no pain and 10 meaning the most severe pain possible. The overall perceived exertion during labor was measured using the adapted Borg scale for perceived effort [25,26]. This scale is graded from 6 to 20, starting with 6 (no feeling of exertion) and ending with 20 (very, very hard). A score of 11–14 indicates moderate activity (fairly light to somewhat hard), while vigorous activities (hard to very, very hard) are represented by 15 or higher.

### Data Analysis

Data were analyzed using GraphPad InStat 3.0 (GraphPad Software Inc.: San Diego, CA, USA). The mean ± standard deviation, frequency, and percentage were used to represent the data. Two-sample t-tests and Wilcoxon sum rank tests were used to compare continuous data between the control and exercise groups, while a chi-square test was used to compare the categorical data. The null hypothesis was rejected at *p* > 0.05. Graph-Pad Instat 3.0 (GraphPad Soft-ware: San Diego, CA, USA).

## 3. Results

### 3.1. Demographic Data

Out of the 158 subjects who agreed to participate in the study, six were excluded due to incomplete data, leaving 76 participants in each group. The average age of subjects in the control and exercise groups was 25.80 and 26.10 years, respectively (Table 2). There were no statistically significant differences in age, height, BMI, or job details among the subjects between the two groups (*p* > 0.05). At least 33% and 32% of the subjects in the exercise and control groups, respectively, reported to follow an active lifestyle before becoming pregnant. In the exercise group, 39% of the subjects reported to have completed post-graduate education, while 17% reported senior secondary or lower. On the other hand, in the control group, 26% were postgraduates, while 26% reported senior secondary or lower (*p* < 0.05).

### 3.2. Nature and Duration of Exercises in the Control and Experimental Groups

Details about the exercises performed by the subjects in the control and experimental groups are provided in Table 3. At least 47% of the subjects in the control group reported to have walked for 6 months to full term, while 66% subjects in the exercise group reported to have followed supervised exercises, including or excluding yoga, for the same duration. The majority of the subjects in both the groups reported walking or exercising 1–3 times per week with sessions lasting for half an hour. In the exercise group, 8% of the subjects reported performing only exercises, 3% only yoga, 11% engaged in walking in addition to exercises, 69% engaged in walking in addition to yoga, and 11% engaged in walking, exercises, and yoga.

### 3.3. Obstetrical Data

*For the course of labor and delivery* (Table 4): 22% of the subjects in the exercise group, compared to 49% in the control group, needed labor induction (*p* < 0.05). The cesarean section rate was 37% in the exercise group compared to 95% in the control group. In total, 63% of subjects in the exercise group delivered vaginally compared to 5% of the subjects in the control group (*p* < 0.05). The mean duration of delivery in the exercise group was 401.05 min, while that in the control group was 607.45 min (*p* < 0.05).

*Maternal weight gain and newborn infant weight* (Table 4): The average weight gain from 12–14 weeks of gestation to 6 weeks after delivery was 11.5 kg in the exercise group, which was significantly lower than the 15.1 kg in the control group (*p* < 0.05). The mean newborn birth weight was significantly higher in the exercise group (3156.6 g) compared with the control (2905.5 g) group (*p* < 0.05).

*Post-partum recovery* (Table 4): The time to resume household tasks and return to one’s job (if employed) was significantly shorter in the exercise group (86%) compared with the control (67%) group (*p* < 0.05).

*Back pain during pregnancy* (Table 5): All the subjects included in this study reported feeling back pain at some point during their pregnancy. However, the mean score of their worst pain on the VAS was significantly lower in the exercise group (6.5 points) compared with the control (8.0 points) group (*p* < 0.05).

*Pain and overall discomfort during labor* (Table 5): The results indicated that the labor pain score reported by the subjects in the exercise group (7.5 points) was significantly lower than that in the control group (9 points) (*p* < 0.05). However, there was no statistically significant difference in the overall discomfort perceived by the subjects during labor between the groups.

## 4. Discussion

This retrospective study was performed to assess the effects of antenatal exercises, including yoga, on the course of labor, delivery, and pregnancy outcomes. The results showed that the subjects who followed antenatal exercises, including yoga, had a lower rate of cesarean section, lower weight gain, higher newborn infant weight, lower pain and overall discomfort during labor, lower back pain throughout pregnancy, and earlier post-partum recovery.

According to the American College of Obstetricians and Gynecologists (ACOG), pregnant women should exercise for at least 30 min on most days of the week [5,27,28]. However, this recommendation is not widely followed due to the various concerns that exercise at this stage may lead to maternal or fetal injury [29,30]. Only a small number of pregnant women (21% in Ireland, 20% in Spain, and 16% in the USA) have been reported to exercise according to the ACOG guidelines [31,32,33]. For most of these women, it was difficult to find correct advice, motivation, and family/community support to begin regular exercise [34,35,36]. Inconclusive evidence on the benefits and risks of exercise for the mother and her fetus is another reason [22].

The results of studies showing the effects of exercises on the rate of cesarean section seem to vary with the type of exercise and its dosage [11]. There was no effect from light resistance and toning exercises done for 3 days a week on the mode of delivery [37]. On the other hand, women who did 40 min of moderate intensity exercise regularly throughout their pregnancy had a significantly lower rate of cesarean section [38]. It has also been reported that inactive women are 3.7 times more likely to have a cesarean section than active women who engage in at least 30 min of moderate physical activity every day [21]. According to a Danish National Birth Cohort study, active women have a 40% lower risk for premature births than those who do no exercises at all [39]. Primiparous women have three times higher risk of having a cesarean section than multiparous women [31]. The rate of cesarean section has also been shown to depend on the healthcare facility where the delivery is conducted, as well as population characteristics including BMI [11].

In the current study, the cesarean section rate in the exercise group was 37% compared with 95% in the control group. Nine subjects reported last minute changes in the infant’s position from cephalic to breech, increased blood pressure, increased bilirubin, no dilatation, a large infant head, unbearable pain, intrahepatic cholestasis during pregnancy, meconium, and prolonged labor as the reasons to opt for cesarean section. Our results were consistent with other studies showing that exercise intervention for more than 50 h throughout the pregnancy can offer a greater reduction in risk for cesarean section [11,40,41,42,43]. In the exercise group, 65% of the subjects performed exercises throughout the pregnancy, 42% of the subjects did exercise 35 times per week, and for 24% of the subjects, exercise sessions lasted for at least half an hour to one hour.

At least 92% of the subjects in the exercise group reported to perform some form of yoga in addition to walking or other forms of exercises. In the last two to three decades, yoga has been extensively used as a technique to prevent and treat various diseases worldwide [44,45]. Yoga is a non-invasive and non-pharmacological method that has been shown to improve strength and flexibility [46]. The regular practice of yoga has been shown to be beneficial for both mind and body, for various reasons, including increased spinal flexibility, improved circulation of cerebrospinal fluid, and enhanced release of endorphins and serotonin [47,48]. It also has the capacity to raise the threshold of pain perception [4,17]. Yoga can strengthen and increase the flexibility of the perineal, vaginal, and urinary sphincter muscles and may thus facilitate labor and delivery through an increase in pelvic diameters [49]. It can help mothers tune their bodies to the consequences of labor by increasing muscle tone, energy, and relaxation [45,50]. It can also improve maternal posture and strengthen the muscles of the back, abdomen, and pelvis that are stressed during labor [45,50,51]. These could also be the reasons for the lower rates of labor induction, shorter durations of delivery, and lower self-perceived labor pain in the exercise group.

Although it has been argued that physical exercise can reduce the infant birthweight and thereby reduce the risk of cesarean delivery [22,33], our results showed that the newborn weight was significantly higher (approximately 250 g) in the exercise group. Other studies have also shown that performing exercises, including yoga, during pregnancy can significantly increase infant weight [4,17]. It has also been reported that exercise augments placental growth during the early and middle periods of pregnancy [52]. The exact phenomenon behind this finding remains unclear.

The overall maternal weight gain during pregnancy was significantly higher in the control (15.1 kg) group compared to the exercise (11.5 kg) group. As mentioned earlier, as the risk of cesarean delivery is positively correlated with BMI [53], the BMI could be another explanation for the lower rate of cesarean delivery in the exercise group. Furthermore, lower weight gain during pregnancy can be beneficial for women’s health in the long term. Post-partum recovery to perform daily activities was significantly faster among the subjects in the exercise group. This could be due to the lower rate of cesarean section, as well as the lower weight gain during pregnancy. Weight gain during pregnancy is normal and is usually due to increased fat and muscle mass, as well as water retention [54]. However, the inability to lose weight after delivery may make the recovery process slower and cause a delay in returning to a normal daily routine.

The nine-month period prevalence of back pain among pregnant women was reported to be as high as 49% [55]. This pain has been shown to prevent such women from performing their daily activities and being physically active [56]. It has also been related to back problems before pregnancy and various physical and psychological factors [57,58]. Although all the subjects reported suffering back pain at some point during their pregnancy, the overall self-perceived pain intensity was significantly lower in the exercise group. The various previous studies showing that exercises during pregnancy can help reduce the intensity of back pain [59,60] support our findings. Yoga improves bodily posture and strengthens the back and abdominal muscles [50], which can in turn decrease back pain [46]. Promoting health alongside other personal values may not only facilitate the introduction of healthy behaviors, but could also reduce several adverse pregnancy outcomes [61].

This study did not consider the effects of exercises on the need for an episiotomy or epidural anesthesia, perineal tears, stages of labor, or Apgar score. We recommend developing a prospective study design on a larger sample size that includes various outcome measures to assess the effects of antenatal exercises, including yoga, on various aspects of pregnancy.

## 5. Conclusions

The results of this retrospective study showed that regular antenatal exercises, including yoga, can help to lower the rate of cesarean section, decrease maternal weight gain, increase newborn infant weight, decrease pain and overall discomfort during labor, and lower back pain throughout pregnancy and aid in earlier post-partum recovery. These results indicate that pregnant women should be active throughout their pregnancy and follow a supervised exercise program, including yoga, unless contraindicated. We require a large-scale prospective studies and quasi-experimental trials to confirm the observed findings.

## Figures and Tables

**Table 1:** Questionnaire used in the study.

Number	Question	Response
Demographic information		
1	Age (years)	
2	Height (cm)	
3	Weight at 12–14 weeks of gestation (kg) Weight at 6 weeks after delivery (kg)	
4	Educational level	Senior secondary or lower
		Diploma
		Graduation
		Postgraduation or higher
5	Job details	House wife
		Working
6	Lifestyle before becoming pregnant	Sedentary
		Active
		Very active
Nature and duration of exercises		
1	Did you do any supervised exercises during pregnancy?	Yes
		No
		Only walking
2	Duration of exercises during pregnancy	0–3 months
		3–6 months
		6 months–full term
3	Frequency of exercises	None
		1–3 times/week
		3–5 times/week
		>5 times/week
4	Duration of exercise session	<Half an hour
		Half an hour–one hour
		>One hour
5	Nature of exercises	None
		Walking
		Supervised antenatal exercises (including resistance, aerobic, pelvic floor, stretching, and relaxation exercises)
		Yoga
Obstetrical information		
1	Need for labor induction	Yes
		No
2	Duration of delivery (minutes)	
3	Nature of delivery	Normal vaginal
		Cesarean section If so any specific reason?
4	Newborn infant weight (grams)	
5	Total maternal weight gain (kg)	
6	Post-partum recovery (time taken after delivery to return to household tasks like making bed, sweeping/mopping/cleaning, grocery shopping, kitchen work without help, and return to job if employed)	Early (2–3 weeks)
		Delayed (>3 weeks)
Self-perceived back pain		
1	Did you suffer back pain during pregnancy	Yes
		No
2	If yes, what was your worst pain on VAS, 0–10? 0 = no pain, 10 = maximum pain	
Self-perceived pain and perceived exertion during labor		
1	Self-perceived pain (VAS, 0–10) 0 = no pain, 10 = maximum pain	
2	Overall perceived exertion during labor (adapted Borg scale, 6–20) 6 = no feeling of exertion, 20 = very, very hard	

VAS, visual analog scale.

**Table 2:** Demographic information of the subjects in the control (*n* = 76) and experimental (*n* = 76) groups).

Variables	Control Group	Exercise Group	*p*-Value
Age * (years)	25.80 ± 2.50	26.10 ± 1.98	>0.05
Height * (cm)	170.01 ± 2.36	169.80 ± 2.20	>0.05
Body mass index * (kg/cm^2^)	24.60 ± 2.60	23.90 ± 2.90	>0.05
Educational level#			
Senior secondary or lower	20 (26.31)	13 (17.10)	<0.05
Diploma	15 (19.73)	13 (17.10)	>0.05
Graduation	21 (27.63)	20 (16.31)	>0.05
Postgraduation or higher	20 (26.31)	30 (39.47)	<0.05
Job details#			
House wife	45 (59.21)	44 (57.89)	>0.05
Working	31 (40.78)	32 (42.10)	>0.05
Lifestyle before becoming pregnant#			
Sedentary	48 (63.15)	47 (61.84)	>0.05
Active	24 (31.57)	25 (32.89)	>0.05
Very active	4 (5.26)	4 (5.26)	>0.05

* Mean ± SD, # number (%).

**Table 3:** Nature and duration of exercises in the control (*n* = 76) and experimental (*n* = 76) groups.

Variables	Control Group Number (%)	Exercise Group Number (%)
Duration of exercises during pregnancy		
0–3 months	28 (36.84)	2 (2.63)
3–6 months	12 (15.78)	24 (31.57)
6 months–full term	36 (47.36)	50 (65.78)
Frequency of exercises		
None	8 (10.52)	0 (00)
1–3 times/week	52 (68.42)	30 (39.47)
3–5 times/week	8 (10.52)	32 (42.10)
>5 times/week	8 (10.52)	14 (18.42)
Duration of exercise session		
<Half an hour	44 (54.89)	32 (42.10)
Half an hour–one hour	20 (26.31)	26 (34.21)
>One hour	12 (15.78)	18 (23.68)
Nature of exercises		
None	20 (26.31)	0 (00)
Only walking	56 (73.68)	0 (00)
Only supervised antenatal exercises	0 (00)	6 (7.89)
Only yoga	0 (00)	2 (2.63)
Walking + yoga	0 (00)	52 (68.42)
Walking + supervised antenatal exercises	0 (00)	8 (10.52)
Yoga + supervised antenatal exercises	0 (00)	0 (00)
Walking + supervised antenatal exercises + yoga	0 (00)	8 (10.52)

**Table 4:** Obstetrical information of the subjects in control (*n* = 76) and experimental (*n* = 76) groups.

Variables	Control Group	Exercise Group	*p*-Value
Need for labor induction#			
Yes	37 (48.66)	17 (22.36)	<0.05
No	39 (51.31)	59 (77.63)	<0.05
Nature of delivery#			
Normal vaginal	4 (5.26)	48 (63.15)	<0.05
Cesarean section	72 (94.73)	28 (36.84)	<0.05
Duration of delivery * (minutes)	607.00 ± 45.03	401.50 ± 50.01	<0.05
Maternal weight gain * (kg)	15.10 ± 1.60	11.50 ± 2.10	<0.05
Newborn infant weight * (grams)	2905.50 ± 350.10	3156.60 ± 420.10	<0.05
Post-partum recovery#			
Early	51 (67.10)	65 (85.52)	<0.05
Delayed	25 (32.89)	11 (14.47)	<0.05

* Mean ± SD, # number (%).

**Table 5:** Score of back pain (VAS) during pregnancy, labor pain (VAS), and overall discomfort level (adapted Borg scale) during labor in the control (*n* = 75) and experimental (*n* = 75) groups.

Variables	Control Group Mean ± SD	Exercise Group Mean ± SD	*p*-Value
Self-perceived back pain during pregnancy	8.00 ± 1.00	6.50 ± 1.50	<0.05
Self-perceived labor pain	9.00 ± 1.00	7.50 ± 1.50	<0.05
Overall discomfort level during labor	17.00 ± 1.00	16.00 ± 1.00	>0.05

VAS, visual analog scale.

## Abbreviations

VAS	Visual analog scale
IUGR	Intrauterine growth retardation
BMI	Body mass index
ACOG	American College of Obstetricians and Gynecologists
